# Psychometric validation of the food and nutrition literacy scale (Escala de Literacia da Alimentação e Nutrição – *E*-LAN) among Portuguese youth

**DOI:** 10.1016/j.pmedr.2026.103392

**Published:** 2026-01-27

**Authors:** Maria João Batalha, Camila Rosinha, Catarina Amaro, Mariana Couto, Mariana Fidalgo, Sara Simões Dias

**Affiliations:** a ciTechCare – Center for Innovative Care and Health Technology, Polytechnic of Leiria, Portugal; b ENSP – Escola Nacional de Saúde Pública – Universidade Nova de Lisboa, Portugal; c School of Health Sciences, Polytechnic of Leiria, Portugal

**Keywords:** Food literacy, Nutrition literacy, Health promotion, *E*-LAN, Psychometric validation, Portuguese youth

## Abstract

**Objectives:**

Psychometric Validation of the *Escala de Literacia da Alimentação e Nutrição* (*E*-LAN), addressing the current lack of validated instruments for assessing food and nutrition literacy among portuguese youth.

**Study design:**

Cross-sectional.

**Methods:**

The study was conducted in a school setting in the western region of central Portugal between April 10 and 12, 2024, using a convenience sample of children aged 10–12 years. The *E*-LAN comprises 49 items (42 Likert-scale and 7 multiple-choice) across seven subscales assessing cognitive and skills domains. Reliability was examined using Cronbach's α, and confirmatory factor analysis (CFA) was performed to evaluate the internal structure, using χ^2^/df, AGFI, CFI, TLI, and RMSEA as fit indices.

**Results:**

The initial scale showed good internal consistency (Cronbach's α = 0.875). Four items were removed due to low item-total correlations and factor loadings. The revised 38-item version demonstrated improved reliability (Cronbach's α = 0.889), acceptable inter-item correlations, and good model fit (χ^2^/df = 1.45; AGFI = 0.91; CFI = 0.93; TLI = 0.91; RMSEA = 0.06).

**Conclusions:**

The *E*-LAN is a concise, reliable, and valid tool for assessing food and nutrition literacy among portuguese youth, supporting its use in public health research and interventions.

## Highlights

• The scale assesses youth food and nutrition literacy.
• The Portuguese version includes 38 Likert items and 7 multiple choice items.
• The scale showed good internal consistency and acceptable six-factor fit.
• Its application provides data to guide public health programs.

## 1 Introduction

Overweight and obesity are major public health issues in the WHO European Region (WHO regional Office for Europe, 2022), requiring integrated approaches that emphasize education and health literacy to support informed health choices (Huang et al., 2024; Friis et al., 2016). Health literacy is essential for supporting healthier choices, and research emphasizes the importance of food literacy and nutrition literacy as integral parts of this broader concept (Truman et al., 2020). Food and nutrition literacy are closely related yet conceptually distinct. Nutrition literacy focuses on understanding and applying nutritional information, while food literacy is broader, encompassing practical application and environmental, economic, and social aspects of the food system (Truman et al., 2017; Vidgen and Gallegos, 2014). The integrated food and nutrition literacy construct combines nutritional knowledge and practical competencies, offering a robust framework to guide public health and nutrition education interventions (Doustmohammadian et al., 2022; Buyinza et al., 2025). Higher food and nutrition literacy in adolescents is associated with healthier diets and better health outcomes, while low literacy is linked to inadequate nutrition and negative physical and psychological effects (Buyinza et al., 2025; Li et al., 2022). Pediatric age is a key period for establishing lifelong healthy habits, highlighting the importance of food and nutrition education (Birch et al., 2007; Vaitkeviciute et al., 2015). In a systematic review by Carroll et al., 12 tools were identified for measuring nutrition literacy and/or food literacy in children and adolescents (Carroll et al., 2022). One of the instruments identified is the Food and Nutrition Literacy (FNLIT) scale, originally developed in Iran for adolescents aged 10 to 12 years. It was later translated into Portuguese and cross-culturally adapted for use with adolescents aged 10 to 19 years, resulting in the *Escala de Literacia da Alimentação e Nutrição* (*E*-LAN). To our knowledge, no validated Portuguese-language instrument currently exists that comprehensively assesses food and nutrition literacy in this age group, making the *E*-LAN a novel tool within the Portuguese context. Nevertheless, the *E*-LAN has not yet undergone full psychometric validation (Cidade Coelho et al., 2022). Thus, this study aims to validate the psychometric properties of the E-LAN to ensure that it consistently and accurately measures the constructs for which it was developed.

## 2 Methods

### 2.1 Study design and population

Data were collected at a school from 5th to 12th grade, in the western region of central Portugal. A convenient sample of 165 participants was obtained. The questionnaire was administered in person to five classes using a guided reading approach. Participation required guardian consent, and all data were collected anonymously and confidentially between April 10 and 12, 2024. The study followed institutional ethical guidelines and was approved by the Ethics Committee of the Polytechnic Institute of Leiria (IPLeiria; reference no. 29/2024).

The inclusion criteria for participants in this study consisted of children and adolescents aged between 10 and 12 years (5th and 6th grades) who demonstrate proficiency in understanding spoken and/or written Portuguese. The exclusion criteria encompassed children with special educational needs that hinder their ability to complete the questionnaire independently, as well as participants with incomplete responses in the *E*-LAN scale. All remaining participants were included in the final sample.

### 2.2 Participants

The sample comprised 83 students, including 28 (33.7%) from 5th grade and 55 (66.3%) from 6th grade, with slightly more boys (54.2%, *n* = 45) than girls (45.8%, *n* = 38). Most mothers (96.2%) and fathers (87.3%) completed higher education. These results are shown in Table 1.

**Table 1:** Demographic and educational characteristics of Leiria study participants (Leiria, Portugal; survey conducted April 10–12, 2024).

n (%)		
Grade	Grade 5th	85 (51.50%)
	Grade 6th	80 (48.50%)
	Total	165 (100%)
Sex	Male	86 (52.10%)
	Female	79 (47.90%)
	Total	165 (100%)
Mothers' education level	Basic Education	33 (22.10%)
	Secondary Education	69 (46.30%)
	Higher Education	47 (31.50%)
	Total	149 (100%)
Fathers' education levels	Basic Education	48 (33.10%)
	Secondary Education	54 (37.20%)
	Higher Education	43 (29.70%)
	Total	145 (100%)

### 2.3 Measures

The *E*-LAN consists of 49 items, comprising 42 Likert scale response items and 7 multiple choice items. These items are distributed across seven subscales: (1) *understanding of food and nutrition information*, (2) *knowledge of the impact of diet on health*, (3) *functional food and nutrition literacy*, (4) *interactive food and nutrition literacy*, (5) *food choice literacy*, (6) *critical food and nutrition literacy*, and (7) *food labeling literacy*. Subscales 1 and 2 assess the cognitive domain, while the remaining subscales evaluate the skills domain. The percentage score for each subscale and the overall scale is calculated as follows: Percentage Score = [(subscale or scale score – minimum possible score on the subscale or scale) / (maximum possible score on the subscale or scale - minimum possible score on the subscale or scale)] × 100. Each response item on a Likert scale is scored from 1 to 5, while multiple-choice response items are scored as either 1 or 5. The percentage score is further categorized into low level for scores ≤51%, moderate level between 51% and 74%, and high level for scores ≥74%.

### 2.4 Statistical analysis

The psychometric elements of the *E*-LAN were examined in accordance with the COSMIN checklist (Mokkink et al., 2024). Subscale 7 was excluded from the psychometric study due to differing question types, with 42 Likert scale responses being analyzed. Data quality was assessed by mean, median and number of missing data. The reliability of the scale was evaluated through internal consistency, Cronbach's alpha (α), the mean inter-item correlations, and the corrected item-total correlation. An α value exceeding 0.70 is deemed acceptable (Pallant, 2020; Streiner et al., 2015). The mean inter-item correlations should fall within the range of 0.15 to 0.5, ensuring that the items collectively measure the same construct while minimizing redundancy (Clark and Watson, 2019). Furthermore, each item should exhibit a corrected item-total correlation with the overall construct greater than 0.30 (Boateng et al., 2018). To validate the internal structure of the questionnaire, a confirmatory factor analysis (CFA) was conducted to assess whether the observed data fit the theoretical model of the subscales proposed in the original Iranian version. For CFA, participants with missing data in any of the questions from 1 to 42 were excluded from the analysis using a listwise deletion approach, and the Weighted Least Squares Mean and Variance Adjusted (WLSMV) estimation method was applied. Goodness of fit was evaluated using multiple criteria: the chi-square to degrees of freedom ratio (X^2^/df), Comparative Fit Index (CFI), Tucker-Lewis Index (TLI), Adjusted Goodness of Fit Index (AGFI), and Root Mean Square Error of Approximation (RMSEA). A good fit is indicated by an X (Huang et al., 2024)/df ratio of 2 or lower. CFI, TLI, and AGFI values of 0.90 or higher reflect a well-fitting model, while an RMSEA value of 0.10 or below is considered acceptable (Marôco, 2014). Items with factor loadings below 0.30 were re-evaluated, as these loadings are generally considered unacceptable, suggesting that the item has low relevance to the latent factor (Hair et al., 2005). *t*-test and Welch's t-test were conducted to compare *E*-LAN scores between boys and girls, as well as between 5th and 6th grade students. The level of statistical significance was set at *p* < 0.05.

For the CFA, R software version 4.4.2 was used. All other analyses were conducted using the Statistical Package for the Social Sciences (SPSS) version 28. The CFA model plot was built in JASP version 0.19.1.

## 3 Results

### 3.1 Data quality

Items 14, 18, and 20 were slightly skewed to the left, while the remaining items were slightly skewed to the right. The response rate for the items was high, with items 2 and 16 having the highest number of missing data (3).

### 3.2 Reliability and construct validity

**Fig. 1:**
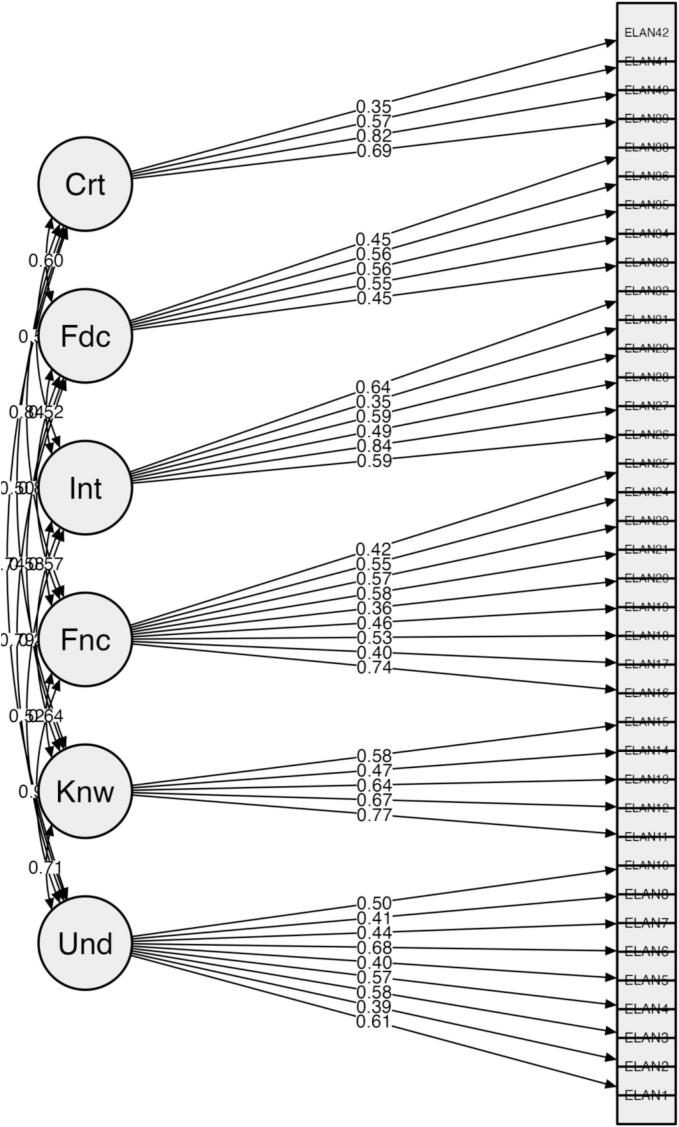
Model plot of the 38-item E-LAN scale. Notes: E-LAN = Escala de Literacia da Alimentação e Nutrição, Und = Understanding, Kwn = Knowledge, Fnc = Functional, Int = Interactive, Fdc = Food choice, Crt = Critical. All factor loadings are standardized.

The overall scale demonstrated good internal consistency with a Cronbach's α of 0.875. Subscale reliability coefficients ranged from 0.53 to 0.71 (Table 2). Specifically, within the cognitive domain, the (1) *understanding* subscale showed acceptable reliability (α = 0.71), while the (2) *knowledge* subscale exhibited moderate reliability (α = 0.67). In the skills domain, the (3) *functional* (α = 0.72) and (4) *interactive* (α = 0.69) subscales demonstrated acceptable to moderate reliability, whereas the (5) *food choice* (α = 0.60) and (6) *critical* (α = 0.55) subscales showed lower reliability. The average inter-item correlation was 0.16, falling within the theoretically acceptable range. The corrected item-total correlations ranged from −0.115 (item 30) to 0.613 (item 16), with items 9, 14, 20, 22, 28, 30, 31, 37 and 42 showing corrected item-total correlations below 0.30, suggesting weak alignment with the construct being measured. Removing items 9, 30, and 37 would increase Cronbach's α. The results of CFA revealed that items 9, 30, and 37 had factor loadings below 0.30, indicating low relevance to their respective latent factors. The fit indices for the 42-items *E*-LAN scale, presented in Table 3, demonstrate a good fit for the factorial structure. Items 9, 30, and 37 were removed for failing statistical criteria, and item 22 for lacking theoretical relevance. After removing these four items, Cronbach's α for the remaining 38 items increased to 0.889, higher than the original 42-item version. The initial version of the *E*-LAN is provided in the supplementary material. The α values for the subscales in this revised version ranged from 0.554 to 0.717 (Table 2). Items 14, 28, 31, and 42 had low item-total correlations (<0.30), with only item 31's removal increasing Cronbach's α. The mean inter-item correlation for the revised *E*-LAN was 0.18, which continues to fall within the acceptable range. The fit indices for the model, after item removal, are reported in Table 3, demonstrating a good fit for the factorial structure comprising the six subscales and two domains. Fig. 1 displays the standardized factor loadings for the factor model in the construct validity study of the revised *E*-LAN (38 items).

**Table 2:** Cronbach's α for the 42-item and 38-item *E*-LAN scales and their subscales among participants in the Leiria study (Leiria, Portugal; survey conducted April 10–12, 2024; *n* = 148).

		Number of items	Cronbach's α	Number of items	Cronbach's α
E-LAN (original version)		42	0.88	38	0.89
Cognitive domain	Understanding	10	0.69	9	0.71
	Knowledge	5	0.67	5	0.67
Skills domain	Functional	10	0.71	9	0.72
	Interactive	7	0.53	6	0.69
	Food choice	6	0.52	5	0.60
	Critical	4	0.55	4	0.55

Notes: E-LAN = Escala de Literacia da Alimentação e Nutrição.

**Table 3:** Confirmatory factor analysis fit indices for the 42-item and 38-item E-LAN scale models among participants in the Leiria study (Leiria, Portugal; survey conducted April 10–12, 2024; n = 148).

Model	Χ^2^/gl	CFI	TLI	AGFI	RMSEA
38-item	1.39	0.83	0.82	0.90	0.05
42-item	1.45	0.84	0.83	0.91	0.06

Notes: E-LAN = Escala de Literacia da Alimentação e Nutrição, X (Huang et al., 2024)/df = Chi-square to degrees of freedom ratio, CFI = Comparative Fit Index, TLI = Tucker-Lewis Index; AGFI = Adjusted Goodness of Fit Index; RMSEA = Root Mean Square Error of Approximation.

### 3.3 Percentage score of the participants

Following psychometric validation and item removal, percentage scores were calculated for 148 students who completed all 38 items. Among them, 3.60% exhibited low levels of food and nutrition literacy, 47.90% exhibited moderate levels, and 38.20% exhibited high levels. Only 19.60% had high scores in E-LAN skill domain, whereas 47.30% attained high scores in the cognitive domain. Subscale 7 had the highest performance, with 87.80% at a high level, whereas subscale 4 had most students (34.50%) at a low level. Subscale 3 also showed concerning results, with over 72.00% scoring moderate or low. Girls scored statistically higher than boys on (3) *functional*, (5) *food choice* subscales and Total. When comparing 5th and 6th grade students, differences were found in (3) *functional* subscale, with 5th grade students showing better results. Details are shown in Table 4.

**Table 4:** Comparison of E-LAN scores between male and female students and between 5th and 6th grade students in the Leiria study (Leiria, Portugal; survey conducted April 10–12, 2024; n = 148).

	Male	Female	*p*-value		Grade 5th	Grade 6th	p-value	
	Mean ± SD	Mean ± SD	Unilateral	Bilateral	Mean ± SD	Mean ± SD	Unilateral	Bilateral
Understanding	67.85 ± 12.25	70.24 ± 11.86	0.11	0.21	68.72 ± 11.98	69.30 ± 12.26	0.38	0.76
Knowledge	78.57 ± 11.84	80.31 ± 11.88	0.18	0.35	80.65 ± 12.84	78.10 ± 10.63	0.09	0.17
Functional	58.74 ± 15.01	67.33 ± 15.58	<0.01	<0.01	65.10 ± 16.00	60.50 ± 15.42	0.03	0.07
Interactive	57.19 ± 16.81	59.62 ± 16.89	0.18	0.36	59.34 ± 16.22	57.33 ± 17.50	0.23	0.45
Food choice*	66.22 ± 17.57	74.11 ± 11.71	<0.01	<0.01	69.53 ± 17.59	70.50 ± 13.06	0.34	0.69
Critical	61.05 ± 19.92	63.56 ± 16.98	0.20	0.39	63.86 ± 18.62	60.55 ± 18.49	0.13	0.26
Food labeling	92.69 ± 14.82	90.42 ± 17.53	0.18	0.37	90.92 ± 16.47	92.32 ± 15.90	0.29	0.58
Total	69.15 ± 9.95	72.24 ± 10.56	0.04	0.07	71.28 ± 10.79	70.03 ± 9.91	0.23	0.46

*t*-tests were used to compare scores between boys and girls, and between 5th- and 6th-grade students; *Welch's *t*-test was applied when equal variance assumptions were not met.

Notes: E-LAN = Escala de Literacia da Alimentação e Nutrição, SD = Standard deviation.

## 4 Discussion

To our knowledge, this study represents the first effort to validate a food and nutrition literacy scale specifically for Portuguese youth aged 10 to 12 years. The *E*-LAN includes 49 items, comprising 42 Likert scale items and 7 multiple choice items, organized into 7 subscales, only the Likert scale items were included in the psychometric analysis. A similar approach was employed in validating the FNLIT scale, where exploratory factor analysis supported a six-factor structure, and CFA indicated an acceptable model fit. Subscale reliabilities ranged from α = 0.48 to 0.80, although the overall Cronbach's α was not reported. In *E*-LAN, subscale α values ranged from 0.52 to 0.71, with the overall scale demonstrating good internal consistency (α = 0.87). Subscales with fewer items showed lower Cronbach's α values, reflecting the variability observed in the item scores; however, their relevance was confirmed by an expert panel during the translation and cross-cultural adaptation process carried out by Coelho et al (Cidade Coelho et al., 2022; Stump and Eng, 2018)Concerning the item-total correlations, several items in the *E*-LAN (9, 14, 20, 22, 28, 30, 31, 37, and 42) exhibited weak correlations (<0.30), indicating poor alignment with the construct. Notably, removing items 9, 30, and 37 would improve Cronbach's α, suggesting their potential exclusion could enhance reliability. Consistently, CFA revealed that these same items (9, 30, and 37) exhibited factor loadings below 0.30, underscoring their low relevance to their respective latent factors. The overall fit indices for the model suggested a good factorial structure across all subscales (Table 3). Item 9 demonstrated low factor loadings and corrected item-total correlations, likely reflecting its limited alignment with the intended food and nutrition literacy constructs. Item 30 showed weak alignment with the construct, likely because children's food choices are heavily influenced by parental habits and feeding strategies, independent of their literacy level (Scaglioni et al., 2018). Finally, item 37 may be irrelevant in the Portuguese context, as all products in commercial spaces are properly displayed and stored. Thus items 9, 30, and 37 were. It was also decided to eliminate item 22, as, similarly, to item 9, it is not directly related to the constructs that the *E*-LAN scale aims to measure. Regarding items 14, 20, 28, 31, and 42, it was decided to retain them in the E-LAN scale, as they exhibited factor loadings greater than 0.30 in the CFA and were considered theoretically relevant. After removing items 9, 22, 30, and 37, Cronbach's α increased compared to the initial. Most subscales showed internal consistency above 0.70, except subscales 5 and 6, likely due to their small number of items, as they contain only five and four items, respectively (Stump and Eng, 2018). Similar findings have been reported by Doust Mohammadian et al. and Ashoori et al (Ashoori et al., 2020; Doustmohammadian et al., 2017) Items 14, 28, 31, and 42 continued to show corrected item-total correlations below 0.30. The CFA of the revised *E*-LAN confirmed item suitability, with all factor loadings above 0.30 and fit indices indicating a strong six-subscale, two-domain structure. The model fit indices were acceptable but marginal, which may be attributed to item redundancy (i.e., some items may assess overlapping aspects of the same construct), ceiling and floor effects in the items (when responses cluster at scale extremes, e.g., “always” or “never”), and the relatively small sample size, which may compromise the stability and precision of parameter estimates (Jakobsson, 2023; Savalei and Huang, 2025). Compared to the original Iranian version, AGFI (0.91) and Χ^2^/df (1.45) improved, while RMSEA remained consistent (0.06). *E*-LAN demonstrated strong internal consistency (α = 0.87), supporting its reliability and use for assessing food and nutrition literacy in Portuguese youth. The results obtained with the E-LAN can be interpreted within the framework of Nutbeam's hierarchical model of health literacy. Specifically, subscales 1–3 reflect functional literacy, encompassing the basic comprehension and application of nutrition information. Subscales 4–5 correspond to interactive literacy, capturing the application of knowledge in real-life contexts and the ability to engage with peers, family, and professionals to promote healthy dietary choices. Finally, subscales 6–7 represent critical literacy, assessing the capacity to critically appraise nutrition information and make informed decisions that influence both personal health and the wider food environment (Savalei and Huang, 2025; Nutbeam, 2008; Yuen et al., 2018). The validation of the *E*-LAN scale for Portuguese youth (10–12 years) reveals both similarities and differences compared to other food and nutrition literacy instruments. The Spanish Short Nutrition Literacy Scale (S-NutLit), targeting young adults (18–25 years), is shorter (11 items) and focuses on two subscales (information skills and expert skills), demonstrating good internal consistency (α = 0.79–0.83) and satisfactory test-retest reliability (Vrinten et al., 2023). The Hungarian adaptation of the Short Food Literacy Questionnaire (SFLQ) also showed high internal consistency (α = 0.85) and factorial validity, though with a unidimensional structure, in contrast to the multifactorial structure of *E*-LAN (Keczeli et al., 2025). These comparisons suggest that while core literacy constructs are preserved, variations in item number, factorial structure, and target age groups reflect contextual and cultural differences that should be considered when interpreting results and applying the scales across populations.

In this study, 3.60% of participants revealed low levels of food and nutrition literacy, higher than Coelho et al.'s findings, likely due to the younger age range (10–12 vs. 10–18 years) (Cidade Coelho et al., 2022). Compared to Iranian adolescents using the original FNLIT scale, this study's sample had fewer participants with low food and nutrition literacy levels (3.60% vs. 11.60%) and more with high literacy levels (42.60% vs. 23.90%) (Doustmohammadian et al., 2020). Consistent with Iranian results, skills domain scores were lower than cognitive domain scores, indicating that knowledge is not always applied in practice. This aligns with the bibliometric analysis conducted by Silva, reinforcing the urgency of further research to identify interventions that are truly effective in enhancing literacy, ultimately leading to healthier and more sustainable food choices (Silva, 2023). Subscale 7 achieved the highest scores in Portugal, whereas Iran showed significantly low scores. This contrast may be attributed to Iran's recent implementation of mandatory nutritional labeling and existing gaps in regulatory policies, along with the lack of integration of the concept into public education programs (Doustmohammadian et al., 2020). Conversely, in Portugal, understanding food label information is firmly embedded in the 6th grade curriculum, underscoring a significant difference between the two contexts (Bonito et al., 2013).

Research on food and nutrition literacy in this age group is limited, with existing scales not providing comparable data. Further studies are essential to understand literacy levels, facilitate cross-country comparisons, and inform public health interventions aimed at promoting healthier eating habits.

The scale's main strength is its translation and cultural adaptation to Portuguese, based on Nutbeam's hierarchical health literacy model (Nutbeam, 2008). The initial Portuguese adaptation involved a qualitative pre-test with semi-structured interviews to ensure item clarity (Cidade Coelho et al., 2022). This study added value to the instrument by providing a quantitative validation using a new independent dataset, with CFA and item analyses confirming the adequacy of the model. However, the scale also presents some limitations. Firstly, it is a lengthy instrument (approximately 45 min to complete), which hinders the assessment of reproducibility through repeated administration to the same individuals. The lack of a gold standard limited assessment of convergent validity, which future studies could address through correlation analyses with related variables. The use of self-reported data can lead to social desirability bias, as participants might give responses, they think are more socially acceptable instead of truthful accounts of their knowledge or behaviors (Camerini and Schulz, 2018). Additionally, the sample was drawn from a single school, which may limit its representativeness and restrict the generalizability of the results to the broader Portuguese youth population. Finally, cultural nuances, stemming from family dietary practices, nutrition education contexts, and local sociocultural environments, may have subtly influenced how certain items were interpreted.

## 5 Conclusion

In conclusion, *E*-LAN shows acceptable internal consistency, factor loadings above 0.30, and good fit indices, confirming its suitability for assessing food and nutrition literacy in Portuguese youth. The scale can be applied in school curricula and public health monitoring to inform and evaluate interventions promoting food and nutrition literacy and healthier behaviors. Future research should validate the instrument in more diverse populations and through longitudinal studies to strengthen evidence on the development of food and nutrition literacy among Portuguese youth.

## CRediT authorship contribution statement

**Maria João Batalha:** Writing – original draft, Methodology, Formal analysis, Conceptualization. **Camila Rosinha:** Writing – review & editing, Investigation. **Catarina Amaro:** Writing – review & editing, Investigation. **Mariana Couto:** Writing – review & editing, Investigation. **Mariana Fidalgo:** Writing – review & editing, Investigation. **Sara Simões Dias:** Writing – review & editing, Supervision, Project administration, Methodology, Formal analysis, Conceptualization.

## Ethics approval and consent to participate

This study received ethical approval (reference (Vrinten et al., 2023)/2024) from the Ethics Committee of the Polytechnic Institute of Leiria (IPLeiria). Participation was limited to youth whose legal guardians provided informed consent.

## Declaration of generative AI and AI-assisted technologies in the writing process

During the preparation of this work the authors used ChatGPT in order to improve the English of the article, as it is not their native language. This tool was also utilized to help summarize certain paragraphs. After using this tool/service, the authors reviewed and edited the content as needed and take full responsibility for the content of the publication.

## Funding

This work was funded by Portuguese national funds provided by Fundação para a Ciência e Tecnologia (FCT) (UI/05704/2025) and by project 2023.04951.BDANA with https://doi.org/10.54499/2023.04951.BDANA identifier.

## Declaration of competing interest

The authors declare that they have no known competing financial interests or personal relationships that could have appeared to influence the work reported in this paper.

## Data Availability

The data supporting this study's findings were obtained from the ATIVA+Saúde project and consist of anonymized information collected from youth in a school setting. Due to ethical considerations and consent restrictions, the dataset is not publicly available.

## Appendix A Supplementary data

Supplementary material: Supplementary data to this article can be found online a (to be inserted). Image 1
