# Supplementary material for: Psychometric validation of the food and nutrition literacy scale (Escala de Literacia da Alimentação e Nutrição – E-LAN) among Portuguese youth
Source: Prev Med Rep. 2026 Jan 27;62:103392. doi: 10.1016/j.pmedr.2026.103392 (PMC12877818; doi:10.1016/j.pmedr.2026.103392)
Supplement: Supplementary file 1 — Supplementary material: Supplementary data to this article can be found online a (to be inserted). [file mmc1.docx]

**Table S1: Confirmatory Factor Analysis: Residuals for the 42-Item E-LAN Scale (n = 148)**

Notes: E-LAN = Escala de Literacia da Alimentação e Nutrição

**Escala de Literacia da Alimentação e Nutrição (E-LAN)**

Escala de Literacia da Alimentação e Nutrição (E-LAN) para alunos do 2.º e 3.º Ciclo do Ensino Básico e Ensino Secundário.

**Por favor, lê com atenção todas as questões e afirmações que se seguem e, para cada uma delas, seleciona (com um X) apenas uma das opções.**

1. **No momento da compra, quão importante é para ti a informação nutricional que consta na rotulagem dos alimentos e bebidas?**

Nada importante Pouco importante Neutro Importante Muito importante

1. **No momento da compra, quão importante é para ti as embalagens dos diferentes alimentos e bebidas terem todas o mesmo tipo de rótulos?**

Nada importante Pouco importante Neutro Importante Muito importante

1. **No momento da compra, quão importante são para ti as datas de produção e validade dos alimentos e bebidas?**

Nada importante Pouco importante Neutro Importante Muito importante

1. **Sou capaz de compreender com facilidade a informação nutricional presente nas embalagens dos alimentos e bebidas (ex.: valor energético, açúcar, proteína, entre outros).**

Nunca Raramente Às vezes Frequentemente Sempre

1. **Sou capaz de compreender com facilidade conteúdos relacionadas com nutrição que leio em jornais, revistas, folhetos.**

Nunca Raramente Às vezes Frequentemente Sempre

1. **Sou capaz de compreender as recomendações alimentares dos nutricionistas relacionadas com necessidades nutricionais e de saúde apropriadas à minha idade.**

Nunca Raramente Às vezes Frequentemente Sempre

1. **Cozer é um dos métodos de culinária mais saudáveis.**

Discordo plenamente Discordo Não concordo nem discordo Concordo Concordo plenamente

1. **Sou capaz de compreender informações e recomendações acerca de alimentação e nutrição adequada às crianças e adolescentes nos meios de comunicação social (ex.: TV, Internet, Rádio, entre outros).**

Nunca Raramente Às vezes Frequentemente Sempre

1. **A prática de 30 a 40 minutos de atividade física diária previne a obesidade.**

Discordo plenamente Discordo Não concordo nem discordo Concordo Concordo plenamente

1. Sei como são cultivados e obtidos os diferentes hortícolas (ex.: cenoura, brócolos, couve-flor).

Não sei nada Sei pouco Sei assim assim Sei bem Sei muito bem

1. **O consumo de snacks salgados (ex.: batatas fritas, rissóis e croquetes) faz mal à saúde.**

Discordo plenamente Discordo Não concordo nem discordo Concordo Concordo plenamente

1. **O consumo excessivo de açúcar, doces e chocolates faz mal à saúde.**

Discordo plenamente Discordo Não concordo nem discordo Concordo Concordo plenamente

1. **O consumo de produtos processados com alto teor de gordura (ex: chouriço, salpicão) pode causar obesidade.**

Discordo plenamente Discordo Não concordo nem discordo Concordo Concordo plenamente

1. **O consumo de produtos processados com alto teor de gordura pode causar cancro.**

Discordo plenamente Discordo Não concordo nem discordo Concordo Concordo plenamente

1. **Ler a informação relativa às datas de produção e validade dos alimentos e bebidas é importante para a saúde.**

Discordo plenamente Discordo Não concordo nem discordo Concordo Concordo plenamente

1. **Eu como pelo menos 2 hortícolas diferentes todos os dias.**

Nunca Raramente Às vezes Frequentemente Sempre

1. **Eu partilho com os outros (ex.: amigos, família, entre outros) informação sobre alimentação e nutrição que obtenho a partir de diversas fontes.**

Nunca Raramente Às vezes Frequentemente Sempre

1. **Converso com os meus amigos e família acerca de alimentação saudável.**

Nunca Raramente Às vezes Frequentemente Sempre

1. **Quando tenho dúvidas relativas a questões sobre alimentos e nutrição consigo obter informação e conselho dos meus pais, professores, entre outros.**

Nunca Raramente Às vezes Frequentemente Sempre

1. **Eu preparo os meus próprios lanches para levar para a escola.**

Nunca Raramente Às vezes Frequentemente Sempre

1. **Eu levo lanches saudáveis para a escola.**

Nunca Raramente Às vezes Frequentemente Sempre

1. **Eu faço exercício físico pelo menos 3 vezes por semana (ex.: jogar futebol, nadar, dançar) ou caminho 60 minutos todos os dias.**

Nunca Raramente Às vezes Frequentemente Sempre

1. **Eu lavo e preparo fruta e hortícolas.**

Nunca Raramente Às vezes Frequentemente Sempre

1. **Eu como fruta todos os dias.**

Nunca Raramente Às vezes Frequentemente Sempre

1. **Eu tomo o pequeno-almoço todos os dias.**

Nunca Raramente Às vezes Frequentemente Sempre

1. Eu tenho força de vontade suficiente para resistir a comer alimentos pouco saudáveis (ex.: fast-food, pizza, **refrigerantes, gelados, chocolates entre outros).**

Nunca Raramente Às vezes Frequentemente Sempre

1. **Se eu for a um restaurante com os meus amigos e todos eles escolheram comida pouco saudável (ex.: pizza, batatas fritas, refrigerantes, entre outros), consigo optar por comida saudável.**

Nunca Raramente Às vezes Frequentemente Sempre

1. **Eu consigo facilmente dizer “Não” a qualquer proposta para consumir comida pouco saudável por parte dos meus amigos.**

Nunca Raramente Às vezes Frequentemente Sempre

1. **Sou capaz de resistir a alimentos e bebidas pouco saudáveis quando me deparo com eles em casa, na escola ou em outros contextos.**

Nunca Raramente Às vezes Frequentemente Sempre

1. **Eu aceito quando os meus pais ou família preparam lanches pouco saudáveis (ex.: batatas fritas, gomas de fruta, bolos, sumos) para eu levar para a escola.**

Nunca Raramente Às vezes Frequentemente Sempre

1. Se na minha família se verificassem casos de excesso de peso ou de uma dieta rica em gordura, eu dir**lhes-ia para mudar os seus hábitos alimentares.**

Nunca Raramente Às vezes Frequentemente Sempre

1. **Quando vou às compras em família, temos a preocupação de comprar lanches saudáveis tais como nozes, fruta e pão em vez de batatas fritas, bolos, chocolates e doces.**

Nunca Raramente Às vezes Frequentemente Sempre

1. **Quando vou às compras em família, temos a preocupação de comprar alimentos que contenham alegações nutricionais (ex.: rico em Ferro, rico em proteína, com baixo teor de gordura, entre outros).**

Nunca Raramente Às vezes Frequentemente Sempre

1. **Quando vou às compras em família, temos a preocupação de comprar alimentos com rótulos.**

Nunca Raramente Às vezes Frequentemente Sempre

1. **Quando vou às compras em família, tenho em consideração o prazo de validade.**

Nunca Raramente Às vezes Frequentemente Sempre

1. **Quando vou às compras em família, temos a preocupação de comprar alimentos e bebidas com embalagens sustentáveis (ex.: embalagens de cartão ou plástico reutilizável).**

Nunca Raramente Às vezes Frequentemente Sempre

1. **Quando vou às compras em família, temos a preocupação de comprar apenas alimentos e bebidas adequadamente expostos em exposição e venda.**

Nunca Raramente Às vezes Frequentemente Sempre

1. **Todos os dias, como alimentos dos 7 grupos de alimentos presentes na Roda dos Alimentos (Cereais e derivados, tubérculos; Hortícolas; Fruta; Lacticínios; Carne, pescado e ovos, Leguminosas; Gorduras e óleos).**

Nunca Raramente Às vezes Frequentemente Sempre

1. **Eu provo com frequência alimentos novos.**

Nunca Raramente Às vezes Frequentemente Sempre

1. **Eu provo com frequência hortícolas novos.**

Nunca Raramente Às vezes Frequentemente Sempre

1. **Dependendo do dinheiro que tenho, eu consigo comprar alimentos saudáveis na escola ou na sua proximidade.**

Nunca Raramente Às vezes Frequentemente Sempre

1. Tenho dificuldade em optar por um lanche saudável se na escola ou na sua proximidade não existir oferta **de alimentos saudáveis.**

Nunca Raramente Às vezes Frequentemente Sempre

1. **Alguma vez viste a informação nutricional numa embalagem de alimentos ou bebidas?**

Sim Não

1. **Para o seguinte par de rótulos alimentares, escolhe o que achas mais saudável.**

| \| **DECLARAÇÃO NUTRICIONAL** \| \|  \| \| --- \| --- \| --- \| \| **VALORES MÉDIOS** \| **POR 100G DE PRODUTO** \| **%VDR** \| \| Energia \| 152 kcal \| 7,60% \| \| Lípidos \| 4,83 g \| 6,9% \| \| Dos quais ácidos gordos saturados \| 3,2 g \| 9,2% \| \| Dos quais ácidos gordos trans \| 0 g \| - \| \| Hidratos de Carbono \| 14,8 g \| 5,69% \| \| Dos quais açúcares \| 0,5 g \| 0,5% \| \| Proteínas \| 12,4 g \| 24,8% \| \| Sal \| 1,1 g \| 18,3% \| | \| **DECLARAÇÃO NUTRICIONAL** \| \|  \| \| --- \| --- \| --- \| \| **VALORES MÉDIOS** \| **POR 100G DE PRODUTO** \| **%VDR** \| \| Energia \| 52,9 kcal \| 2,7% \| \| Lípidos \| 3,2 g \| 4,6% \| \| Dos quais ácidos gordos saturados \| 2 g \| 10% \| \| Dos quais ácidos gordos trans \| 0 g \| - \| \| Hidratos de Carbono \| 2,88 g \| 1,1% \| \| Dos quais açúcares \| 0,36 g \| 0,4% \| \| Proteínas \| 3,15 g \| 6,3% \| \| Sal \| 0,1 g \| 1,7% \| |
| --- | --- | --- | --- | --- | --- | --- | --- | --- | --- | --- | --- | --- | --- | --- | --- | --- | --- | --- | --- | --- | --- | --- | --- | --- | --- | --- | --- | --- | --- | --- | --- | --- | --- | --- | --- | --- | --- | --- | --- | --- | --- | --- | --- | --- | --- | --- | --- | --- | --- | --- | --- | --- | --- | --- | --- | --- | --- | --- | --- | --- | --- |

**RÓTULO ALIMENTAR A RÓTULO ALIMENTAR B**

Rótulo A Rótulo B Não sei

1. **Responde às seguintes questões tendo em conta a informação recolhida relativa a um alimento.**

|  | **Por 100 g** |
| --- | --- |
| Açúcar | 8,8 g |
| Gordura total | 0,9 g |
| Gordura saturada | 0,08 g |
| Sal | 0,215 g |

**Legenda:**

|  | Alto |  | Médio |  | Baixo |
| --- | --- | --- | --- | --- | --- |

- 1. **Como descreverias a quantidade de açúcar neste produto?**

Baixo Médio Alto Não sei

- 1. **Como descreverias a quantidade de gordura total e de gordura saturada neste produto?**

Baixo Médio Alto Não sei

- 1. **Como descreverias a quantidade de sal neste produto?**

Baixo Médio Alto Não sei

1. **Responde às seguintes questões tendo em conta a informação recolhida sobre um pacote de leite pasteurizado.**

|  | **Porção de 240 mL de leite** |
| --- | --- |
| Energia | 86 kcal |
| Açúcar | 0 g |
| Gordura total | 0,4 g |
| Gordura saturada | 0 g |
| Sal | 0,3 g |

**Legenda:**

|  | Alto |  | Médio |  | Baixo |
| --- | --- | --- | --- | --- | --- |

- 1. **Como descreveria a quantidade total de gordura neste produto?**

Baixo Médio Alto Não sei

- 1. **Como descreveria a quantidade de sal neste produto?**

Baixo Médio Alto Não sei
